# A Predictive Model for the Migration and Bioaccumulation of Residual Malachite Green in Aquaculture Sediments: Simulation and Validation

**DOI:** 10.3390/toxics14090794

**Published:** 2026-09-07

**Authors:** Shengnan Zhang, Xizhuang Zhu, Xi Chen, Liping Qiu, Shunlong Meng, Xingxing Fang, Chao Song

**Affiliations:** 1 Wuxi Fisheries College, Nanjing Agricultural University, Wuxi 214081, Chinamengsl@ffrc.cn (S.M.); 2 Freshwater Fisheries Research Center, Chinese Academy of Fishery Sciences, Wuxi 214081, China; 3 Laboratory of Quality & Safety Risk Assessment for Aquatic Products on Environmental Factors (Wuxi), Ministry of Agriculture and Rural Affairs, Wuxi 214081, China; 4 Key Laboratory of Control of Quality and Safety for Aquatic Products, Ministry of Agriculture and Rural Affairs, Beijing 100141, China; 5 College of Fisheries, Huazhong Agricultural University, Wuhan 430070, China; 6 College of Animal Science & Technology, Nanjing Agricultural University, Nanjing 210095, China; 7 Sanya Institute of Nanjing Agricultural University, Nanjing 572025, China

**Keywords:** malachite green, leucomalachite green, mandarin fish, bottom sediment, bioaccumulation, aquatic product safety

## Abstract

Aquatic product safety is closely linked to human health, yet the effects of historical residual drugs in aquaculture pond sediment remain unclear. Malachite green (MG), a prohibited aquaculture drug, is rapidly converted to leucomalachite green (LMG) after illegal use and may persist in bottom sediment, raising the question of whether residue-positive sediment can cause positive detections in fish at harvest. In order to answer this question, we developed a time-dependent predictive model for the sediment–water–fish system based on a food web bioaccumulation model and a sediment–water equilibrium model. The model examines the relationship between historical MG residues in sediment and positive detections in aquatic products, and estimates LMG residue levels in fish at harvest. The reliability of this model was further validated through comparison with measured data. The results showed that when the sediment residue concentration was 227.48 μg/kg, the maximum LMG concentration in fish during the culture period reached 0.56 μg/kg, indicating a positive correlation with fish concentration. The closer the habitat water layer was to the sediment, the higher the risk of positive detection in fish; in the middle water layer, the LMG concentration in fish could reach 0.28 μg/kg, and, under ideal conditions, a two-fold relationship existed between the middle and lower water layers. Sediment organic carbon content determined the amount of LMG released into the water; when it was set to 0.0035, the initial LMG concentration in water was 0.07 μg/kg, showing a negative correlation with fish concentration. This study confirmed that a certain amount of residual malachite green in sediment could cause positive detection in mandarin fish during the culture period, which is consistent with the validation experiment. If the model predicted no positive detection at the pollutant concentration corresponding to the sediment of the positive detection pond, this would indicate that malachite green originated from other sources during the culture process; otherwise, the sediment may lead to the risk of positive detection in fish. One limitation of the model is that it may underestimate pollutant concentrations in fish during the early stages of aquaculture. In addition, its ability to predict the effects of combined pollutant exposure remains to be validated. The model developed here can be used to estimate residue levels of aquaculture drugs during the culture period and may provide regulatory authorities with a basis for distinguishing historical residues from illicit use, thereby improving the efficiency of aquatic product quality and safety supervision.

## 1. Introduction

Malachite green (MG) is a synthetic triphenylmethane dye that was once widely used in aquaculture to prevent fungal infections such as water mold and gill mold, as well as certain parasitic diseases [1]. After entering water or fish, MG can be rapidly reduced to leucomalachite green (LMG), which is more lipophilic and therefore more persistent in the environment. Like its parent compound, LMG has been associated with carcinogenic, teratogenic, and mutagenic effects [2].

In response to food safety and environmental protection requirements, many countries have prohibited the use of MG and LMG in aquaculture [3]. In China, MG and LMG are listed as prohibited substances for use in food animals, and prohibited substances are not permitted to be detected in edible animal-derived foods [4]. For aquatic products, the relevant analytical method covers MG and LMG. The malachite green residue indicator is generally expressed as the sum of MG and LMG residues. The value of 0.5 μg/kg was selected with reference to the analytical performance level commonly associated with GB/T 19857-2005 [5]. Therefore, in the present study, 0.5 μg/kg was used as the threshold for a positive result in fish muscle for model interpretation.

Because the model converts MG into LMG for prediction rather than the combined MG and LMG residue, exceedance of this threshold should be interpreted as a screening result based on LMG.

In pond aquaculture systems, once MG enters the environment, it is readily adsorbed by bottom sediment and undergoes migration and transformation among sediment, water, and fish. Sediment therefore serves as an important long-term reservoir for these residues. Previous studies have shown that the dissipation of MG in aquaculture water and sediment is strongly affected by light, disturbance, and environmental conditions [6,7], and that residues in sediment generally dissipate more slowly. At the same time, MG can be rapidly converted to LMG in fish and may persist in different tissues. In mandarin fish (*Siniperca chuatsi*), the distribution and elimination patterns of MGs (malachite green and leucomalachite green) in tissues have been reported [8], suggesting that legacy residues in a residue-positive culture environment may continue to influence fish safety at harvest.

A growing body of evidence indicates that fish are exposed to organic pollutants mainly through water and feed, and that internal body burdens are jointly determined by uptake, elimination, metabolic conversion, and growth dilution [9,10]. For compounds with strong hydrophobicity and long environmental persistence, water concentration alone is insufficient to describe residue behavior in fish accurately.

In the present study, a sediment–water equilibrium model was used to describe the quantitative relationship between sediment and water, whereas a food web bioaccumulation model was used to quantify the relationship between water and fish. By coupling sediment–water partitioning with fish uptake and elimination, the contribution of legacy sediment residues to positive detections in harvested fish can be more clearly interpreted. It is therefore of practical importance to develop a predictive model that links sediment residues, culture duration, and fish residues because such a model can help identify the origin of positive results, distinguish legacy residues from illegal drug use, and support the validation of risk.

At present, studies on MG residues have focused mainly on bath exposure [11,12,13], analytical methods [14,15,16,17], and adsorption and degradation [18,19,20]. However, predictive analyses of whether MG residues in sediment can lead to positive detections in mandarin fish during the culture period remain limited. In particular, when sediment acts as a long-term reservoir and continuously releases contaminants into the water column, a predictive tool that can link sediment residue levels to fish residue levels is urgently needed.

Against this background, the present study used mandarin fish as a model species and, by integrating the migration and transformation processes in the sediment–water–fish system, developed a time-dependent residue prediction model. Published residue elimination data from a residue-positive mandarin fish culture environment [21] were used as external validation data to assess the model’s applicability to temporal changes in MG residues and to positive detection judgments. The findings provide a theoretical basis for assessing the risk of legacy MG residues and for improving the safety regulation of aquaculture products.

## 2. Materials and Methods

This study developed a time-dependent concentration model for MG and LMG in the sediment–water–fish system based on a sediment–water equilibrium model, a food web bioaccumulation model, and aquaculture experimental data. The model treated sediment as the primary pollutant reservoir and simulated the dynamic changes in contaminants in different media during a conventional culture cycle by incorporating release and redistribution processes between sediment and water, as well as uptake, elimination, metabolism, and growth dilution in fish. Because MG is relatively unstable and is rapidly transformed into LMG in the environment and in fish, MG was converted to LMG for simulation purposes to facilitate the calculations.

### 2.1. Model Construction

#### 2.1.1. Sediment–Water Equilibrium Model

Because fish are in direct contact with water, the pollutant concentration in sediment must first be converted to the concentration in water. Here, a sediment–water equilibrium model was used to establish the link between sediment and the aqueous phase [22]. Based on the sediment organic carbon normalized partition coefficient *K*_OC_ (L/kg), *C*_S_ (μg/kg) represents the concentration of pollutants in sedimentary deposits. Using the organic carbon fraction *f*_OC_ of the culture sediment, the concentration of LMG in sediment pore water, *C*_W_, was calculated. The unit of *C*_W_ was μg/L. The formula is as follows:

(1) $$C_{W}\;=\;\frac{C_{S}}{\left(K_{\mathrm{OC}}\;\times {\;f}_{\mathrm{OC}}\right)}$$

For sediment, *K*_OC_ has a clear functional relationship with the *K*_OW_ value of the target pollutant. The concentration of LMG in water, *C*_W_, was thus calculated as the basis for predicting fish residues. The formula is:

(2) $$\log K_{\mathrm{OC}}\;=\;0.989\;\times \;\log K_{\mathrm{OW}}\;+\;0.34$$

#### 2.1.2. Food Web Bioaccumulation Model

**Figure 1:**
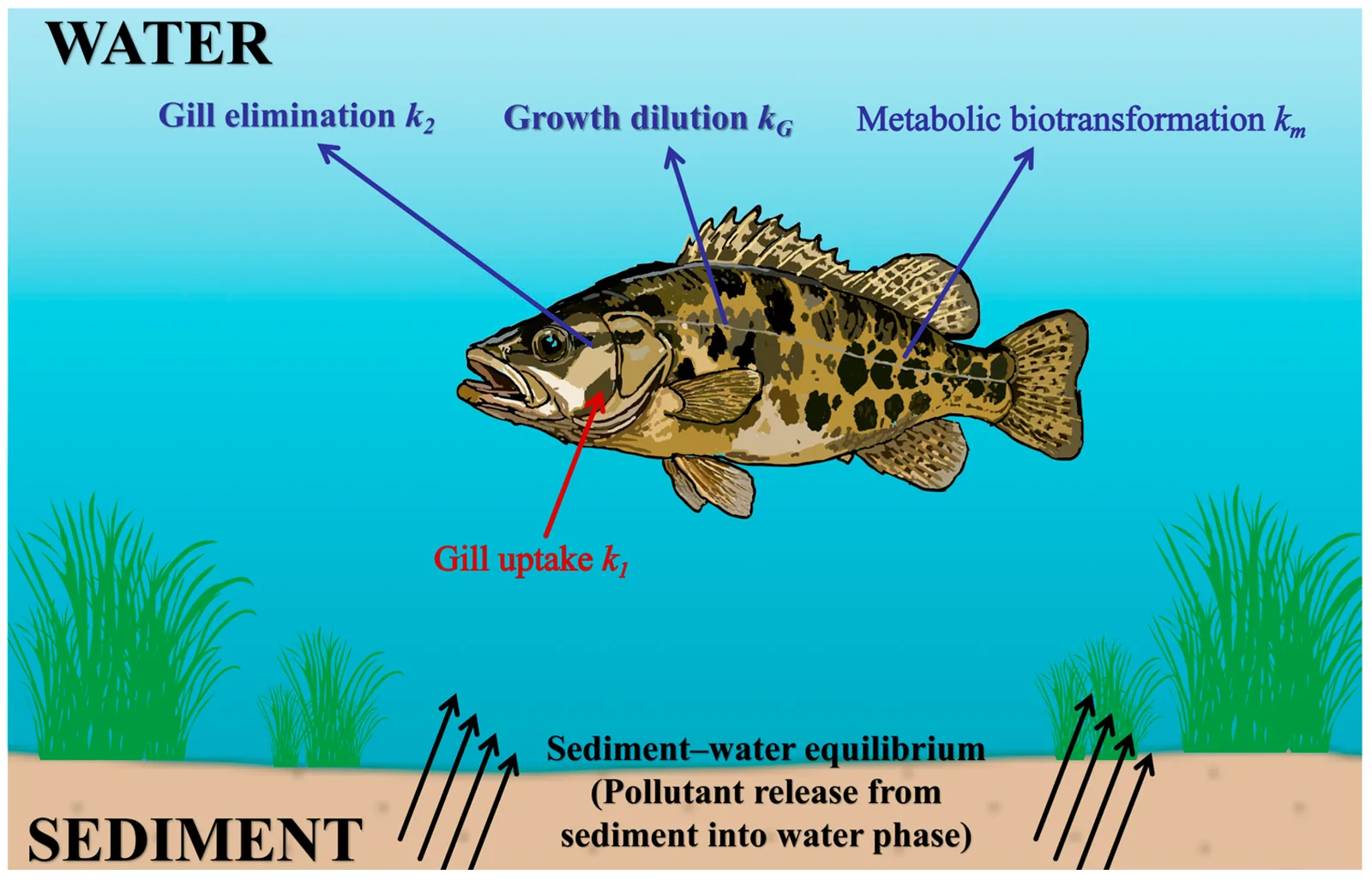
Schematic diagram of sediment–water–fish residue prediction model for leucomalachite green (LMG) in mandarin fish. The red arrow shows the gill uptake (*k*_1_) of LMG from water into fish. Blue arrows represent the elimination pathways from fish: gill elimination (*k*_2_), growth dilution (*k*_G_), and metabolic biotransformation (*k*_m_). Black arrows illustrate sediment-to-water pollutant release under sediment–water equilibrium. These kinetic processes and parameters form the LMG residue prediction model.

Pollutants in sediment first enter the water column by diffusion, and the pathways by which chemicals in water enter fish include gill uptake [23], ingestion, and dermal penetration. Because dermal uptake is minimal [24], it was ignored. As the present study primarily examined the effect of residual LMG in sediment on mandarin fish, uptake through feeding was not considered. Feed or other food containing LMG was also excluded, and only gill uptake was considered. Pollutant elimination from fish mainly occurs through four routes: gill excretion, fecal excretion, growth dilution, and metabolic transformation. Since ingestion was not considered, fecal elimination did not need to be included either. Consequently, the fish concentration at harvest can be expressed as the difference between gill uptake and the combined effects of gill elimination, growth dilution, and metabolic transformation (Figure 1).

Because MG can readily convert to LMG in sediment, water, and fish, and because this process is influenced by temperature, light, oxygen concentration [6,7], and other factors, the half-life of MG in sediment is much shorter than that of LMG [25]. In later simulations, the component of MGs that most strongly affected mandarin fish was mainly LMG. To simplify the calculations and to model long-term changes, the amount of MG in sediment was converted entirely to LMG equivalents. The conversion procedure is described in Section 2.2.

Let *y* (μg/kg) denote the concentration of MGs in mandarin fish at a given time, *C*_S_ (μg/kg) the concentration of LMG in pond sediment at that time, and *C*_W_ (μg/L) the concentration of MGs in water at that time. The rate of change in LMG concentration (*y*) in mandarin fish over time (*t*) is therefore given by:

(3) $$\frac{\mathrm{dy}}{\mathrm{dt}}\;=\;\mathrm{uptake}\;\mathrm{rate}\;-\;\mathrm{elimination}\;\mathrm{rate}$$

The uptake rate of LMG in mandarin fish is a function of the gill uptake rate constant (*k*_1_, L/kg·day), the fish body mass (*m*_f_, kg), and the pollutant concentration in water (*C*_W_). The elimination rate of LMG in mandarin fish consists of three components: the gill elimination rate (*k*_2_, day^−1^), the growth dilution rate (*k*_G_, day^−1^), and the metabolic transformation rate (*k*_m_, day^−1^), together with the instantaneous value of *y* at time *t* [10,26]. These rate parameters together form the fish residue prediction equation. A total of 31 parameters were used in this study, mainly involving pollutant migration and transformation, sediment–water partitioning, fish uptake and elimination, growth dilution, and fish body composition (Table 1).

Organisms that remain in close contact with bottom sediment, such as benthic fish and invertebrates, can exchange chemicals with sediment pore water. Because sediment and water are often not in concentration equilibrium, the concentration of freely dissolved chemicals in pore water may exceed that in the overlying water, and under certain conditions this disequilibrium can be substantial [27].

In this context, *m*_O_ denotes the proportion of respiration using overlying water, whereas *m*_P_ denotes the proportion using sediment pore water. In many cases, benthic fish and invertebrates use little pore water for respiration because oxygen concentration and food availability are low in bottom sediment. Although the proportion of pore water respiration may be small, it can still be important for chemicals under strong disequilibrium between pore water and overlying water. In most cases, *m*_P_ is equal to or less than 5% [28]. For organisms not in direct contact with sediment pore water, *m*_P_ is zero. The relationship between *m*_O_ and *m*_P_ is:

(4) $$m_{O}\;=\;1-m_{P}$$

In the present study, different values of *m*_P_ were used to represent different habitat water layers of mandarin fish. Fish living in the upper water layer were assumed to have negligible contact with sediment, so *m*_O_ = 1.00 and *m*_P_ = 0. Fish living in the middle lower water layer were closer to the sediment and had limited contact with it, so *m*_O_ = 0.95 and *m*_P_ = 0.05. Because excessive stocking may force some fish into the middle lower water layer and increase the time they spend near the bottom, frequent contact with the sediment was represented by *m*_O_ = 0.90 and *m*_P_ = 0.1.

Mandarin fish are demersal fish that commonly remain in burrows during the daytime and emerge to forage at night [29]. Because the conditions considered in this study represent high-density pond culture, some fish may be displaced by crowding and remain close to the pond bottom for extended periods. Therefore, *m_P_* was set to 0.10 for fish inhabiting the bottom layer in the conventional simulation. Because fish may inhabit different water layers within the pond, different *m_P_* values were used to represent the probability of contact between the fish and sediment pore water.

Owing to the high *K_OW_* of LMG, LMG is more likely to be adsorbed by the sediment than to diffuse into the overlying water. In this study, the LMG concentration in the overlying water refers to the concentration of freely dissolved LMG because only the freely dissolved fraction can enter fish through the gills by diffusion, which is consistent with the assumptions of the present model. According to the study by Li et al. [21], MGs were not detected in the water after water exchange, which was conducted 7 days after treatment, with concentrations below the detection limit of 0.1 μg/kg. Therefore, fish inhabiting the upper and middle water layers were expected to have relatively low LMG concentrations. In the extreme case, fish that had no contact with sediment pore water throughout the culture period would have an LMG concentration approaching zero, making it unlikely to reach the detection limit of the analytical instrument. This scenario was therefore also considered in the present study, and *m_P_* was approximately set to zero for the corresponding calculation.

Substituting the above parameters into Equation (4) gives:

(5) $$\frac{\mathrm{dy}}{\mathrm{dt}}\;=\;m_{f}\;\times \;k_{1}\;\times \;(m_{P}\;\times \;C_{W})-(k_{2}\;+\;k_{G}\;+\;k_{m})\;\times \;y$$

where *dy*/*dt* (ng/kg·day) is the rate of change in the pollutant concentration in fish, which in this study refers to the rate of change in LMG in mandarin fish. Based on published growth data, fish body mass and growth time are usually highly exponentially correlated and can be expressed as:

(6) $$m_{f}\;=\;\beta \;\times \;e^{\gamma \;\times \;t}$$

Let:

(7) $$b\;=\;\beta \;\times \;k_{1}\;\times \;C_{W}\;\times {\;m}_{P}$$

and:

(8) $$a\;=\;(k_{2}\;+\;k_{G}\;+\;k_{m})$$

Substituting Equations (7) and (8) into Equation (5) and rearranging yields:

(9) $$\frac{\mathrm{dy}}{\mathrm{dt}}\;+\;a\;\times \;y\;=\;b\;\times \;e^{\gamma \;\times \;t}$$

Integrating Equation (9) piecewise and rearranging gives:

(10) $$\begin{array}{c} y\;=\;\left[\frac{\left(\beta \;\times \;k_{1}\;\times \;C_{W}\;\times \;m_{P}\right)}{\left(k_{2}\;+\;k_{G}\;+\;k_{m}\;+\;\gamma \right)}\right]\;\times \;\left(e^{\gamma \;\times \;t}-e^{-(k_{2}\;+\;k_{G}\;+\;k_{m})\;\times \;t}\right)\; \end{array}$$

However, the available literature does not provide public data for *k*_1_, *k*_2_, *k*_m_, and *k*_G_, so these parameters had to be calculated separately.

#### 2.1.3. Calculation of the Rate Constants *k*_1_, *k*_2_, *k*_m_, and *k*_G_

Calculation of *k*_1_: Arnot and Gobas [26] showed that the gill uptake rate constant (*k*_1_) is the combined result of gill ventilation and chemical absorption across the gills. Furthermore, existing research has demonstrated that fish gill tissue is relatively sensitive to changes in the aquatic environment [30]. Therefore, in fish, invertebrates, and zooplankton, *k*_1_ can be regarded as a function of ventilation rate *G*_V_ (L/day) and gill chemical absorption efficiency *E*_W_ (dimensionless), where m_f_ is organism mass (kg). The formula for *k*_1_ is:

(11) $$k_{1}\;=\;\frac{G_{V}\;\times \;E_{W}}{m_{f}}$$

where *E*_W_ is the gill chemical absorption efficiency and *G*_V_ is the gill ventilation rate (L/day). Gobas [31] proposed that gill chemical absorption efficiency *E*_W_ is a function of the octanol–water partition coefficient (*K*_OW_) of the pollutant, which can be expressed as:

(12) $$E_{W}\;=\;{\left(1.85\;+\;\frac{155}{K_{\mathrm{OW}}}\right)}^{-1}$$

Based on empirical data, Arnot and Gobas reported that fish gill ventilation rate and oxygen consumption are related [26]; specifically, ventilation rate *G*_V_ is associated with dissolved oxygen concentration *C*_OX_, which can be calculated as follows:

(13) $$G_{V}\;=\;\frac{1400\;\times \;{m_{f}}^{0.5}}{C_{\mathrm{OX}}}$$

Here, *C*_OX_ represents dissolved oxygen concentration in the pond (mg/L) and can be expressed as a function of temperature T (°C), calculated using the equation given by Neely [32]:

(14) $$C_{\mathrm{OX}}\;=\;14.45-0.413\;\times \;T+\;0.00556\;\times \;T^{2}$$

In this equation, T (°C) denotes the water temperature in the mandarin fish pond and is determined by the actual culture temperature.

Calculation of *k*_2_: The partitioning of organic chemicals between biota and water is thought to occur among lipids, non-lipid organic matter (NLOM; such as proteins and carbohydrates), and water. For each organism, the biota–water partition coefficient on a wet weight basis (*K*_BW_) is defined as:

(15) $$K_{\mathrm{BW}}\;=\;\nu_{\mathrm{LB}}\;\times \;K_{\mathrm{OW}}\;+\;\nu_{\mathrm{NB}}\;\times \;\delta \;\times \;K_{\mathrm{OW}}\;+{\;\nu }_{\mathrm{WB}}\;=\;\frac{k_{1}}{k_{2}}$$

Thus:

(16) $$k_{2}\;=\;\frac{k_{1}}{k_{\mathrm{BW}}}$$

Here, δ is the proportionality constant representing the adsorption capacity of NLOM for octanol. Based on previous studies [33], δ was set to 0.035. *ν*_LB_, *ν*_NB_, and *ν*_WB_ are the fractions of lipid, NLOM, and water content (kg substance/kg organism), respectively, from which *k*_2_ can be calculated.

Calculation of *k*_m_: *k*_m_ denotes the rate at which compounds are eliminated through metabolic transformation. This process depends on both the chemical and the species. Previous studies [34] indicate that the metabolic transformation rate constant for pollutants in fish is generally 0.00063.

Calculation of *k*_G_: *k*_G_ denotes the growth dilution rate constant. It can be calculated using the formula proposed by Thomann [27]:

(17) $$k_{G}\;=\;0.00251\;\times \;{m_{f}}^{-0.2}$$

(18) $$k_{G}=0.0005\;\times \;{m_{f}}^{-0.2}$$

Equations (17) and (18) are the growth dilution rate constants at 25 °C and 10 °C, respectively. Because the present study was designed to match the growth temperature of mandarin fish, therefore, the growth dilution rate constant at 25 °C is used to approximately describe the growth trend at 22 °C, as specified in this study.

Equations (1)–(18), together with the default value of *k*_m_, jointly define the concentration prediction model used in this study.

### 2.2. Acquisition of the Initial LMG Concentration in Sediment

The initial concentrations of MG and LMG in sediment were determined according to the study by Li [21]. The study showed that after pond water was treated with 0.2 mg/L MG and replaced after 7 d, the residual MG concentration in sediment was 233.48 μg/kg and the residual LMG concentration was 73.38 μg/kg. Because the present study simulated long-term changes over 1–450 d, the decay of both MG and LMG in sediment had to be considered. Since MG is unstable and has a half-life of less than 10 d in sediment [25], and to simplify the long-term simulation, all MG was converted to LMG equivalents for the analysis.

Calculation of the initial LMG content: In addition to conversion to LMG, degradation and elimination of MG also involve direct decomposition. Therefore, the conversion rate from MG to LMG had to be estimated first. In this study, the conversion rate was estimated from the increase in MG and the decrease in LMG within the first MG half-life.

The simulation conditions of the present model assumed that MG was added to the water until its concentration reached 0.2 mg/L. Therefore, the MG half-life equation corresponding to an initial concentration of 0.25 mg/L, as reported in Li et al. [21], was used for the calculations.

(19) $$C_{t}=0.2231\;\times \;e^{\left(-0.0318\;\times \;t\right)}$$

According to Equation (19), the decrease in MG concentration during the first half-life period was approximately 52.33 μg/kg. Over the corresponding period, the increase in LMG concentration was approximately 34.5 μg/kg based on the data reported in that study [25]. The conversion efficiency from MG to LMG was therefore calculated as follows: 34.5 / 52.33 = 66%.

The initial MG and LMG concentrations in the sediment reported in Li et al. [21] were used in the present calculations. Based on the available data, the initial total LMG concentration used in the model was calculated as: 73.38 + 233.48 × 66% = 227.48 μg/kg.

Half-life equation for LMG: The half-life of MG in sediment was 4.14 d [25], whereas the half-life of LMG was much longer than that of MG. The time required for LMG to become non-detectable in all tissues of mandarin fish ranged from 230 to 300 d [8]. Therefore, the half-life of LMG was considered to fall within the range of 4.14–230 days. Half-lives of 45, 90, and 180 days were subsequently selected for comparison (Text S2). The detailed equations and simulation results are provided in the Supplementary Materials (Table S1). To represent an intermediate condition and better reflect the average trend, an LMG half-life of 90 days was selected for the subsequent simulations.

Accordingly, substituting this value into the half-life equation gave *k*_0_ = 0.0077, from which the first order elimination kinetic equation for LMG in sediment was obtained:

(20) $$C_{t}\;=\;227.48\;\times \;e^{\left(-0.0077\;\times \;t\right)}$$

### 2.3. Conventional Culture Simulation for Mandarin Fish and Leucomalachite Green

Mandarin fish and leucomalachite green were selected as the study subjects to explain and apply the model. According to the equations above, the basic data had to be collected and substituted into the model first.

For the fish component of the concentration prediction model, the species, body mass, culture water temperature, and exponential growth equation must be known in advance. For the pollutant component, the species and *K*_OW_ value must be known. In addition, because this study focused on residual pollutants in bottom sediment, the sediment–water equilibrium model also had to be incorporated. For this model, the values of sediment *K*_OC_ and *f*_OC_ in the pond had to be established in order to link pollutant concentrations between sediment and the aqueous phase.

Several representative values were selected for simulation to demonstrate the calculation process. First, the culture water temperature was set to 22 °C. In addition, because fish growth follows a sigmoidal pattern—rapid in the early stage and slower later on, with smaller increases in body mass—the growth data of mandarin fish obtained by Ji et al. [35] from culture experiments were used.

Logistic and other growth equations were initially considered to describe fish growth. Although the logistic growth equation itself can be expressed explicitly, its substitution into the model resulted in an expression that could not be readily rearranged into an analytical form for y. Therefore, this study continues to employ the exponential growth equation for calculations. To reduce the potential overestimation associated with a single exponential model during the later culture period, the study employs two distinct growth equations—one for the pre-growth phase and one for the post-growth phase.

The actual observations from the first 150 d were fitted to an exponential growth equation suitable for the early growth phase (R^2^ = 0.92, *p* < 0.001), as shown in Equation (21). Body mass for days 180–450 was then back-calculated using the Gompertz model with the best R^2^ reported by the authors, and all data from days 1–450 were finally fitted to an exponential growth equation describing the later growth phase (R^2^ = 0.45, *p* < 0.001), as shown in Equation (22):

(21) $$m_{f}\;=\;27.1529\;\times \;e^{0.0152\;\times \;t}$$

(22) $$m_{f}=77.5917\;\times \;e^{0.0071\;\times \;t}$$

Second, for LMG, the available literature indicates that its *K*_OW_ value is 5.72. For the convenience of calculations in this study, a value of 5.70 was adopted [36]. This high *K*_OW_ value indicates strong lipophilicity, which favors accumulation in lipid-rich tissues such as viscera, skin, and muscle [8]. In the present study, the average sediment *f*_OC_ was set to 0.0035, reflecting the organic matter content of a pond immediately after water replacement. The *K*_OC_ value of LMG was then calculated as 9.49 × 10^5^ L/kg.

Because the present study used the published work as the validation basis, the simulated conditions had to be consistent with those in that study (Text S1). An MG-positive aquaculture environment was established by adding technical grade MG to a pond with an area of approximately 1400 m^2^ and a water depth of 1.5 m until the MG mass concentration in the water reached 0.2 mg/L. The water was maintained under these conditions for 7 d [21].

Therefore, in the conventional culture simulation, *f*_OC_ was set to 0.0035 to represent the organic matter content in a pond immediately after water exchange [37]. Substituting the values of *K*_OC_ and *f*_OC_ into the sediment water equilibrium model gave the pollutant concentration in water, *C*_W_ (Table 2).

Calculation of *K*_BW_: Using data from existing studies [38], the lipid fraction, NLOM fraction, and water content (kg/kg) in the mandarin fish body were determined to be 4.36%, 22.00%, and 73.64%, respectively. Substitute the aforementioned basic data into the corresponding rate constant calculation formula to obtain the rate constant, and then substitute both the resulting rate constant and m_P_ into Equation (10) to calculate the *y* value.

### 2.4. Source of External Validation Data

No additional culture experiment was conducted in this study. Instead, the residue elimination results from a residue-positive mandarin fish culture environment reported by Li et al. [21] were used directly as external validation data. The study established an MG-positive culture environment and continuously monitored the elimination of MG and LMG residues in mandarin fish. The dataset can therefore be used to assess the agreement between the model and the actual aquaculture process.

### 2.5. Quantitative Evaluation Metrics

The RMSE, MAE, bias, and R^2^ were calculated using the paired observed and predicted LMG-equivalent concentrations. RMSE and MAE were used to quantify the magnitude of prediction errors, whereas bias was used to assess the direction and magnitude of systematic deviation. The R^2^ value was used to evaluate the agreement between the predicted and observed concentrations. Bias was calculated as the predicted concentration minus the observed concentration; therefore, positive and negative bias values indicate overprediction and underprediction, respectively.

### 2.6. Statistical Analysis

All data processing, model calculations, and statistical analyses were performed using Microsoft Excel 2024 (Microsoft Corporation, Redmond, WA, USA) and OriginPro 2026b (OriginLab Corporation, Northampton, MA, USA). When parameter estimation was required, the model parameters were estimated using nonlinear fitting.

The predictive performance of the model was assessed using the root mean square error (RMSE), mean absolute error (MAE), bias, and coefficient of determination (R^2^). RMSE and MAE were used to quantify the magnitude of prediction errors, bias was used to evaluate the direction and magnitude of systematic deviation, and R^2^ was used to assess the agreement between the predicted and observed concentrations. Lower RMSE, MAE, and absolute bias values, together with a higher R^2^ value, indicated better model performance.

Because the present analysis focused on model prediction and validation rather than hypothesis testing, no inferential statistical tests were performed and no statistical significance threshold was applied.

## 3. Results

### 3.1. Overall Prediction of the Sediment–Water–Fish Residue Model

The compiled data were substituted into Equation (10) to simulate fish residue concentrations under conventional conditions (Table 2). Calculation workflow: Lines 1–12 in the table correspond to the input of basic parameters, lines 13–22 correspond to the equations and parameters used in the calculations, and then the final output is obtained.

**Figure 2:**
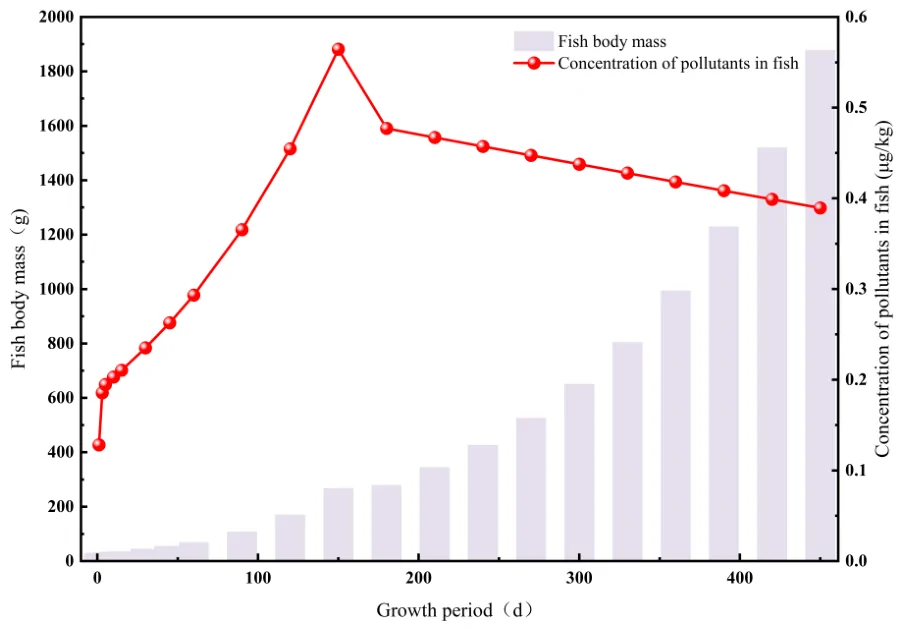
Variation in simulated fish body mass and simulated *y* value (LMG concentration) in mandarin fish over the growth period. Simulated under sediment LMG residue of 227.48 μg/kg, water temperature of 22 °C, and *f*_OC_ = 0.0035. Light purple bars denote simulated fish body mass (left vertical axis); red line with circular symbols represents simulated *y* value (LMG concentration in fish, right vertical axis).

Relationship between simulated body mass and *y* values under conventional conditions. Under the conditions of 0.2 mg/L MG in water for 7 d followed by water replacement, leaving only 227.48 μg/kg of LMG in sediment, a culture water temperature of 22 °C, and *f*_OC_ = 0.0035, mandarin fish still showed positive detection during the culture period (Figure 2). The residue level increased first and then decreased with culture duration and body mass, peaking at around 150 d, when MG concentration in mandarin fish reached 0.56 μg/kg.

The culture period had a significant effect on fish residues. As culture time increased, fish had more opportunities to contact pollutants in the environment, and internal residues were more likely to reach detectable levels. This was especially evident when exposure continued from the early to the later stages of culture, during which residues showed a gradual accumulation trend. After the peak was reached, because the amount of MGs in the environment had decreased substantially, uptake became lower than elimination, and fish body mass continued to increase; consequently, LMG concentration in fish declined during the later culture period.

### 3.2. Effect of Different Initial C_S_ Values on the Positive Detection Risk in Fish

**Figure 3:**
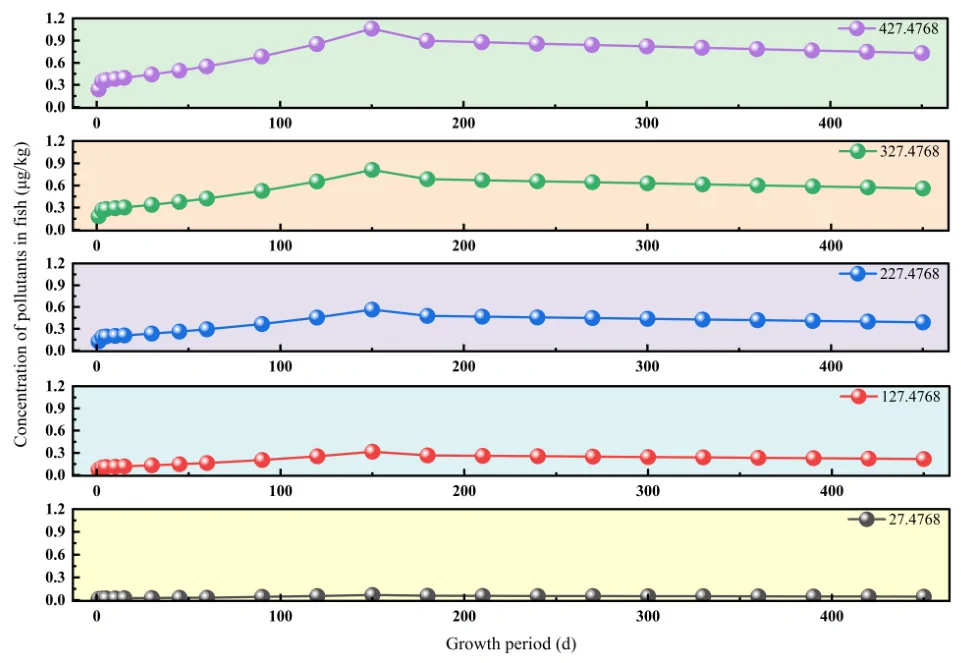
Temporal dynamics of simulated LMG concentration in mandarin fish at different initial sediment LMG concentrations (*C_S_*). From top to bottom, subplots represent sediment LMG concentrations of 427.48, 327.48, 227.48, 127.48, and 27.48 μg/kg. Horizontal axis: growth period (d); vertical axis: LMG concentration in fish (μg/kg).

Effect of different initial *C*_S_ values on *y* values in fish. LMG residue concentrations in mandarin fish increased proportionally with increasing initial *C*_S_ values, consistent with the basic source–sink transport principle for pollutants (Figure 3). The culture water temperature was 22 °C and *f*_OC_ = 0.0035. Additionally, the conventional simulation incorporated an LMG elimination half-life of 90 d. Fish inhabiting the bottom layer, which were assumed to have the most frequent contact with sediment, were selected for the simulation. Because these fish were expected to have the highest probability of positive detection within the pond population, they were used to estimate the upper exposure limit below which positive detection would not be expected. Accordingly, these fish were used to estimate the maximum treatment level that would not be expected to result in positive detection in the fish population.

When sediment contained 27.48 μg/kg of LMG, the maximum LMG concentration in mandarin fish during the culture period was 0.068 μg/kg. When sediment contained 127.48 μg/kg of LMG, the maximum concentration was 0.32 μg/kg. When sediment contained 327.48 μg/kg of LMG, the maximum concentration was 0.81 μg/kg. When sediment contained 427.48 μg/kg of LMG, the maximum concentration reached 1.06 μg/kg.

The model indicated that under conventional simulation conditions, no positive detection would occur during the culture cycle when the initial LMG *C*_S_ was below 200 μg/kg. The maximum LMG concentration in mandarin fish was 0.496 μg/kg, which remained below the positive detection threshold (Table 3).

Sediment acts as a long-term reservoir for MGs, and the metabolite LMG degrades more slowly, entering the water column gradually and continuously. Compared with a model based on water exposure alone, incorporating the sediment residue reservoir introduces cumulative and time-lagged characteristics into the predicted fish residues. It can therefore be inferred that contaminated sediment may represent a potential source of positive detections in harvested aquatic products under actual pond culture conditions.

Moreover, the model indicates that fish residue levels are not determined by a single factor; rather, they are jointly regulated by sediment residue concentration, habitat water layer, culture duration, and sediment organic carbon content. When sediment contamination is high and sediment organic carbon content is low, accumulation becomes more pronounced, and sediment residue concentration becomes the decisive factor. Overall, the model can describe the dynamic evolution of residue risk in cultured fish under sediment contamination.

Legacy residues in culture sediment should not simply be viewed as a remnant of past events; rather, they constitute a realistic source of risk that can directly affect the safety of harvested aquatic products. For drugs such as MG, which is prohibited in aquaculture, environmental persistence and slow release behavior make pond sediment a long-term contamination reservoir [39,40]. Even when no further drug application occurs during culture, residues in sediment can still re-enter the water through environmental redistribution and gradually accumulate in fish, ultimately leading to positive detection.

### 3.3. Effect of Different Habitat Water Layers on the Positive Detection Risk in Fish

**Figure 4:**
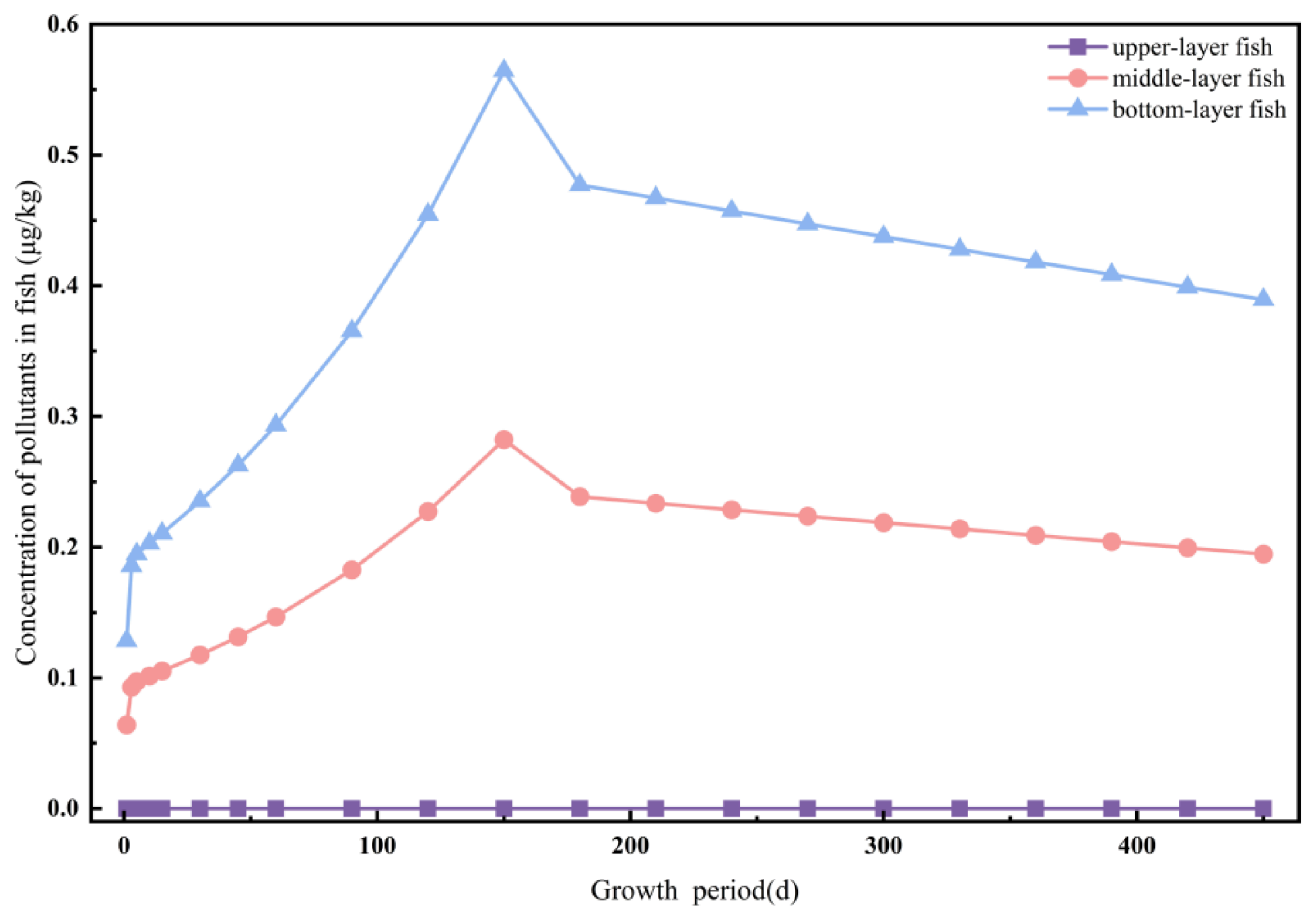
Simulated variation in LMG concentration in fishes inhabiting different water layers over the growth period. The purple square line represents the upper-layer fish; the red circular line denotes the middle-layer fish; and the blue triangular line indicates the bottom-layer fish. The horizontal axis is the growth period (d), and the vertical axis stands for simulated LMG concentration in fish (μg/kg).

Under the same environmental conditions, residue concentration in fish increased with *m*_P_. From highest to lowest, the ranking was bottom-layer fish, middle-layer fish, and upper-layer fish (Figure 4). Since the upper-layer fish’s *m*_P_ is close to 0, the LMG concentration in the upper-layer fish is also close to 0. The maximum LMG concentration in middle-layer fish was 0.28 μg/kg, whereas that in bottom-layer fish reached 0.56 μg/kg. Under idealized conditions, the LMG concentration in middle-layer fish and bottom-layer fish showed a 1:2 relationship. Nevertheless, the influence of water layer distribution on residue concentration was much smaller than that of culture duration, initial sediment concentration, or *f*_OC_.

The effect of habitat water layer on fish residues was mainly due to differences in pollutant concentration across water layers as sediment releases contaminants into the water. The closer the water layer is to the sediment, the higher the pollutant concentration and the greater the amount available for uptake, which in turn increases the probability of positive detection. The model used idealized data for calculation; in reality, fish do not remain in a single water layer [41]. This complexity may further increase the risk of positive detection.

### 3.4. Effect of Different Sediment f_OC_ Values on the Positive Detection Risk in Fish

**Figure 5:**
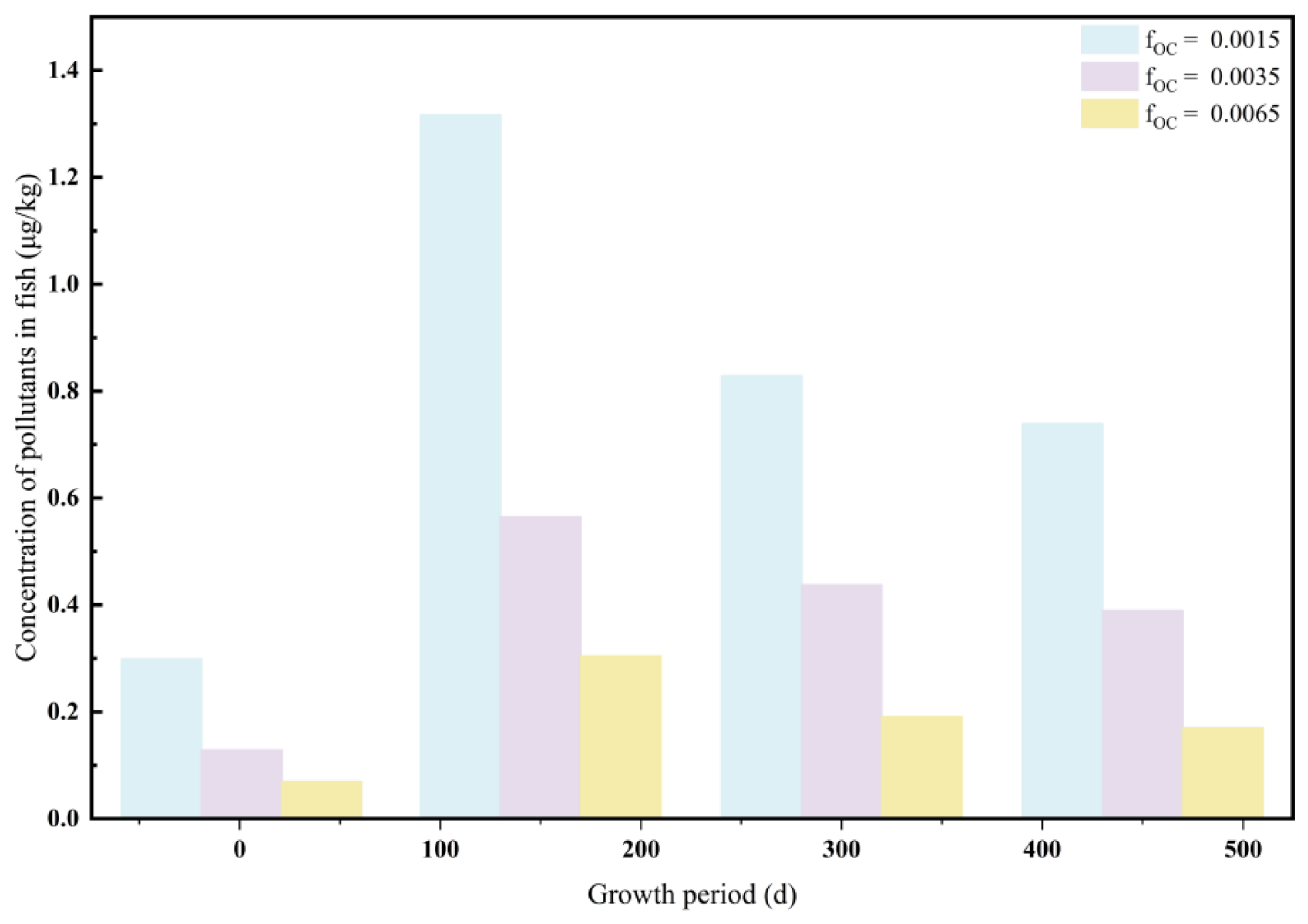
Simulated LMG concentration in mandarin fish under different *f*_OC_ values. Light blue: *f*_OC_ = 0.0015; light purple: *f*_OC_ = 0.0035; and yellow: *f*_OC_ = 0.0065. Horizontal axis: growth period (d); vertical axis: LMG concentration in fish (μg/kg).

LMG residue concentration in mandarin fish gradually decreased as sediment *f*_OC_ increased (Figure 5). Variation in *f*_OC_ did not alter the overall pattern in which LMG concentration first increased and then decreased during the culture period. The lower the *f*_OC_ value, the higher the LMG residue concentration in fish. In pond sediment immediately after dredging and water exchange, the minimum *f*_OC_ was approximately 0.0015, under which the maximum LMG concentration in fish during culture could reach 1.32 μg/kg. The period of high positive detection risk was also markedly prolonged.

Sediment organic carbon content has an important influence on the environmental fate of MG. Differences in organic carbon content alter pollutant partitioning between the solid and liquid phases, thereby affecting release into the water column. Because LMG is a hydrophobic organic compound, it is readily adsorbed by sediment. In general, the lower the sediment organic carbon content, the less likely pollutants are to be retained by sediment, and the higher the risk of positive detection in fish. Conversely, the higher the sediment organic carbon content, the more readily pollutants are adsorbed and fixed by sediment, the slower the transfer to water and fish, and the lower the risk of positive detection [42].

In pond sediment with low organic carbon content, drug residues are more likely to remain in a relatively active release state. Even if the total sediment burden is not particularly high, increased effective release may still lead to fish exposure. By contrast, in sediment with high organic carbon content, environmental bioavailability is relatively reduced, and the risk of positive detection in fish decreases accordingly.

### 3.5. Model Validation and Prediction of Positive Detection at Harvest

To validate the predictive ability of the model, the conditions used in this study were aligned with published data from Li et al. [21]. The validation results showed that under the condition of 0.2 mg/L MG in water maintained for 7 d followed by water replacement, a culture water temperature of 22 °C, and *f*_OC_ = 0.0035, the model predicted that residue levels in mandarin fish could still exceed the 0.5 μg/kg positive detection threshold during a conventional culture cycle. This prediction was consistent with the culture experiment.

According to Li et al. [21], the limits of detection (LODs) for both MG and LMG were 0.1 μg/kg; the reported MG observations were available only up to 70 d, whereas the reported LMG observations were available only up to day 240. At subsequent time points, the results were reported as not detected (N.D.), indicating concentrations below the LOD of 0.1 μg/kg. For model validation, observations below the LOD, including those reported as N.D., were assigned a value of LOD/2, corresponding to 0.05 μg/kg. Because MG was converted to LMG in the present model, the observed MG concentrations were also converted to the corresponding LMG-equivalent concentrations using the MG to LMG conversion efficiency described above. Model validation was then performed by comparing the observed and predicted LMG-equivalent concentrations.

**Figure 6:**
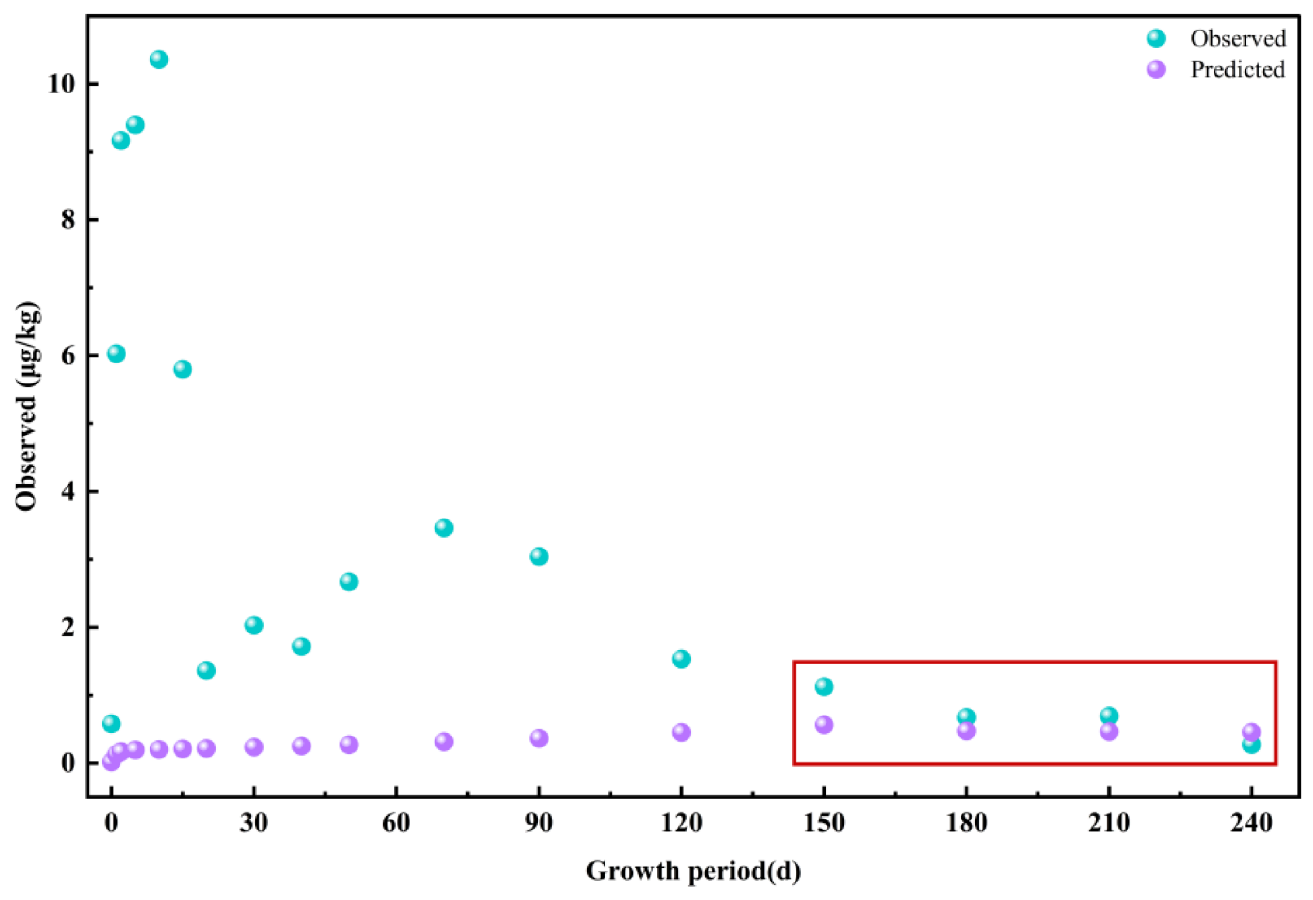
Comparison of the observed LMG-equivalent and predicted concentrations in mandarin fish during the 240 d culture period. The cyan and purple circles represent the observed and predicted concentrations, respectively. The data points enclosed by the red box, corresponding to the period from day 150 to day 240, were used for model validation. Concentrations are expressed in μg/kg.

The scatter plots of the paired observed and predicted values indicated that the model provided relatively accurate predictions during the later culture period, whereas a clear underestimation was observed during the early culture period (Figure 6). Therefore, the observations and corresponding predictions from days 150 to 240 were used for model validation.

During 150–240 d, the model showed moderate agreement with the observed LMG-equivalent concentrations (Table 4), with an R^2^ value of 0.7090. This indicates that the model explained approximately 70.9% of the variability in the observed concentrations during the validation period. The bias was −0.1990, indicating a slight overall underestimation of the observed concentrations. The MAE and RMSE were 0.2911 μg/kg and 0.3299 μg/kg, respectively, indicating that the average absolute prediction error was 0.2911 concentration units and that the root mean square prediction error was 0.3299 concentration units. The higher RMSE than MAE suggests that some prediction errors were larger than the average absolute error, although the difference between the two metrics was relatively limited. Overall, the model provided a reasonable prediction of LMG-equivalent concentrations during the later culture period, but its predictive performance should be interpreted as applicable primarily to after 150 d because the early period observations showed systematic underestimation.

## 4. Discussion

### 4.1. Sediment–Water–Fish Transfer Determines the Temporal Persistence of Residues

The behavior of MG in pond systems is essentially a multi-compartment coupled process. Sediment, as the main reservoir, determines whether contaminants can continue to be released; the water column is the direct medium through which contaminants enter fish; and elimination, metabolism, and growth dilution within fish determine the final detection level. Thus, fish residues are not simply the result of a single exposure event, but rather the product of continuous input, dynamic equilibrium, and gradual accumulation.

The model developed in this study incorporates these processes simultaneously and can therefore more reasonably explain why fish residues may remain relatively high after water replacement. The reason is that, first, elimination of MGs in fish takes time, and LMG in fish only reaches a non-detectable level after 230–300 d [43,44,45,46]; second, although water replacement can temporarily reduce pollutant concentrations in the water column, it cannot completely remove legacy pollutants already adsorbed in sediment. After fresh water is added, pollutants can again enter the aqueous phase as sediment and water re-establish partition equilibrium, thereby continuing to drive exposure in fish. Therefore, relying on short-term water replacement alone is not sufficient to fundamentally eliminate the harvest stage risk caused by sediment contamination.

This finding is important for aquatic product safety supervision. Traditional risk identification usually focuses on whether there has been recent illegal drug use. In contrast, the present study shows that a positive result at harvest does not necessarily imply recent application; it may also originate from sediment residues formed earlier and persisting over time. Accordingly, when judging the cause of a positive result, the historical contamination background should be included in the interpretation framework rather than relying solely on the timing of drug use relative to sampling.

From the perspective of aquaculture management, this means that water replacement alone cannot directly eliminate drug residues in the culture environment. Simply modifying surface management practices without addressing the sediment itself is often insufficient to remove the risk associated with legacy residues. Farmers should also evaluate the physicochemical properties of the sediment and consider measures such as dredging and bottom replacement to better reduce the risk of positive detections in fish. In practical production, it is often difficult to assign responsibility for positive results in a reasonable manner because regulatory authorities cannot easily determine whether the pollutant in fish originated from current illegal addition or from historical sediment residues. Therefore, when new ponds are prepared for culture, measures such as disinfection, dredging, and bottom replacement should be taken [47]. Simply replacing the water is not enough to eliminate historical residues completely.

Based on the time-dependent concentration transfer relationship among sediment, water, and fish, this study developed a residue prediction model for malachite green and its metabolite leucomalachite green in aquaculture systems. The model treats sediment as the main pollutant reservoir [48,49,50,51] and incorporates release and re-partitioning between sediment and water, as well as uptake, elimination, metabolism, and growth dilution in fish, to simulate dynamic changes in pollutant concentration across compartments during a conventional culture cycle.

### 4.2. Analysis of Differences Between the Model Predictions and Experimental Results

Compared with the study by Li [21], the predicted values from this model were somewhat lower, mainly because of the treatment of MG. In the published experiment, after water replacement the sediment still contained 233.48 μg/kg of MG and 73.38 μg/kg of LMG. Because the *K*_OW_ of MG is lower than that of LMG, MG is less readily adsorbed by sediment and therefore can cause a rapid increase in MGs in fish during the early culture stage. In the present study, MG was converted to LMG for long-term simulation and easier calculation, which led to differences in accumulation time.

In addition, the samples in the experiment were not muscle samples in the conventional sense; rather, the head, tail, viscera, and bones were removed and the remaining material was homogenized. Considering the tissue distribution patterns of MGs in mandarin fish, the present study directly used fish body concentration as the positive detection criterion [8,43,44]. Finally, because the water and sediment environment is complex, and because MG entering fish at the beginning undergoes tissue partitioning and metabolite formation, simulated values may fluctuate relative to those observed in actual culture experiments.

### 4.3. Application Potential and Limitations of the Model

The time-dependent concentration prediction model developed here can provide a theoretical quantitative basis for aquaculture management and regulatory responsibility assessment. The model assumes that the sediment is the sole contamination source and is intended to provide supporting evidence for determining whether historical residues in sediment could result in positive detection in fish during the culture period. When the pollutant concentration in the sediment and other relevant environmental parameters are measured and entered into the model, a prediction of no positive detection under the sediment-only contamination scenario, together with an actual positive detection, would provide reasonable grounds for suspecting external contamination. Potential external sources may include unauthorized chemical treatment, contaminated feed, or contaminated juveniles. Further investigation and additional analytical testing could then be conducted to identify the specific cause of the positive detection. Conversely, if the model predicts a positive detection under the sediment-only scenario, the sediment would be considered a likely source, provided that other potential contamination sources have been excluded.

Thus, the model can assist regulatory authorities in source attribution and the assessment of regulatory responsibility. It can also be used to evaluate contamination risks before harvest, thereby helping farmers reduce potential economic losses and shifting risk prevention and control to an earlier stage.

From an application perspective, the model may be used in the following ways. First, pond sediment can be tested before culture begins so that ponds with high legacy residue concentrations are flagged as high risk. Second, during culture, sediment organic carbon content and culture duration can be used to dynamically assess the risk of positive detection at harvest. Third, when product screening results are positive, the model can provide supplementary evidence for determining the source of contamination. Compared with reliance on end point testing alone, this process-based approach to risk identification is more conducive to improving regulatory efficiency and to early warning at the source, shifting aquatic product safety management from post hoc testing to prevention in advance.

At the same time, this study also provides a useful framework for other prohibited or restricted drugs beyond LMG. For compounds with strong environmental persistence and sediment adsorption characteristics, a similar sediment–water–fish dynamic prediction framework may be constructed to perform other pollutant risk assessments.

Although this study can describe the influence of legacy sediment residues on the risk of positive detection in fish, several limitations remain. First, the model parameters were mainly based on literature data and experimental conditions reported in published studies. Actual ponds differ in temperature, sediment composition, dissolved oxygen, and fish physiological state, all of which may affect prediction accuracy. Second, because all MG was converted to LMG in the calculations, the model may underestimate contaminant concentrations in fish tissues during the early culture period. Accordingly, the model showed better predictive performance during the later culture period. In addition, the present study focused mainly on residues of MGs, whereas actual aquaculture environments often contain multiple coexisting drugs or pollutants, and interactions among different substances may further alter exposure and residue behavior. Future work should combine additional field monitoring data to localize model parameters and incorporate multi-pollutant interactions to improve the model’s applicability and explanatory power.

Despite these limitations, by coupling a sediment–water equilibrium model with a fish bioaccumulation model, the present study established a predictive model for pollutant residue concentrations in the sediment–water–fish system. It systematically quantified the effects of culture duration, initial sediment concentration, sediment *f*_OC_, and fish habitat water layer on residue concentration at harvest. The model not only provides a tool for frontline regulators to assess risk and support responsibility attribution, but also contributes to the theory of sediment–water–fish migration and bioaccumulation for prohibited aquaculture drugs, offering theoretical guidance for scientific farming and risk control in mandarin fish production.

## 5. Conclusions

This study demonstrated that a positive culture environment can lead to positive detection in fish at harvest. In addition, the effects of different environmental factors on residue concentrations in fish were also elucidated. In practical risk assessment, the total pollutant concentration in sediment alone cannot be used to determine whether a positive result is likely. Sediment organic carbon content, culture duration, and the habitat water layer of the fish should also be considered. This also helps explain why fish residue levels can differ substantially between ponds even when sediment residue concentrations are similar.

## Figures and Tables

**Table 1:** Key parameters.

Line	Symbol	Definition	Unit
1	a	Pollutant elimination coefficient	day^−1^
2	b	Pollutant accumulation coefficient	μg/kg∙day
3	Bias	Mean bias	μg/kg
4	*C*_OX_	Concentration of dissolved oxygen	mg/L
5	*C*_S_	Pollutant concentration in sediment	μg/kg
6	*C*_W_	Pollutant concentration in water	μg/L
7	*C*_t_	Drug concentration	μg/kg
8	*E*_W_	Chemical uptake efficiency across the gill	Dimensionless
9	*f*_OC_	Organic carbon mass fraction in sediment	g/g
10	*G*_V_	Gill ventilation rate	L/day
11	*k*_0_	First-order elimination rate constant	day^−1^
12	*k*_1_	Gill uptake rate constant	L/kg∙day
13	*k*_2_	Gill elimination rate constant	day^−1^
14	*K*_BW_	Wet-weight bioconcentration factor	Dimensionless
15	*k*_G_	Rate constant for growth dilution	day^−1^
16	*k*_m_	Rate constant for metabolic transformation	day^−1^
17	*K*_OC_	Sediment organic carbon-normalized partition coefficient	L/kg
18	*K*_OW_	Octanol–water partition coefficient	Dimensionless
19	MAE	Mean absolute error	μg/kg
20	*m*_f_	Mass of the fish at t	kg or g
21	*m*_O_	Proportion of respiration using overlying water	Dimensionless
22	*m*_P_	Proportion of respiration using pore water	Dimensionless
23	NLOM	Non-lipid organic matter	Dimensionless
24	R^2^	Coefficient of determination	Dimensionless
25	RMSE	Root mean square error	μg/kg
26	T	Water temperature	°C
27	t	Time	d
28	*y*	Concentration of pollutants in fish	μg/kg
29	*ν*_LB_	Lipid mass fraction in fish muscle	Dimensionless
30	*ν*_NB_	NLOM mass fraction in fish muscle	Dimensionless
31	*ν*_WB_	Water mass fraction in fish muscle	Dimensionless
32	β	Exponential growth constant	Dimensionless
33	γ	Exponential growth constant	Dimensionless
34	δ	Proportionality constant for NLOM adsorption to octanol	Dimensionless

**Table 2:** Calculation workflow.

	Line	Parameter	Units	Results
Inputs	1	*m*_f_	kg or g	Variable
	2	*K*_OW_	Dimensionless	5.70
	3	T	°C	22
	4	*f*_OC_	g/g	0.0035
	5	*k*_m_	day^−1^	0.00063
	6	*C*_S_	μg/kg	227.48
	7	*ν*_LB_	Dimensionless	4.36%
	8	*ν*_NB_	Dimensionless	22.00%
	9	*ν*_WB_	Dimensionless	73.64%
	10	*m*_P_	Dimensionless	0.10
	11	β	Dimensionless	27.1529/77.5917
	12	γ	Dimensionless	0.0152/0.0071
Calculations	13	*C*_OX_	mg/L	8.06
	14	t	d	Variable
	15	*E*_W_	Dimensionless	0.03
	16	*G*_V_	L/day	Variable
	17	*k*_1_	L/kg∙day	Variable
	18	*K*_BW_	Dimensionless	1.03
	19	*k*_2_	day^−1^	Variable
	20	*k*_G_	day^−1^	Variable
	21	*K*oc	L/kg	9.49 × 10^5^
	22	*C*_W_	μg/L	Variable

**Table 3:** *y* values when the initial sediment LMG concentration (*C*_S_) is 200 μg/kg.

**t(d)**	**1**	**10**	**30**	**60**	**90**	**120**
*y* (μg/kg)	0.11	0.18	0.21	0.26	0.32	0.40
**t(d)**	**150**	**180**	**210**	**240**	**270**	**300**
*y* (μg/kg)	0.496	0.43	0.42	0.41	0.40	0.39

**Table 4:** Statistical metrics of model performance during 150–240 d.

Performance Indicator	Value
R^2^	0.7090
Bias	−0.1990
MAE	0.2911
RMSE	0.3299

Note: R^2^, coefficient of determination; Bias, mean bias calculated as the predicted value minus the observed value; MAE, mean absolute error; and RMSE, root mean square error.

## Data Availability

The raw data supporting the conclusions of this article will be made available by the authors on request.

## Supplementary Materials

The following supporting information can be downloaded at https://www.mdpi.com/article/10.3390/toxics14090794/s1, Text S1. The methods of conventional culture simulation; Text S2. The half-life equation of LMG; Table S1. Residual LMG concentrations in sediment and fish tissues under different half-life scenarios. *C*_S_ (μg/kg) denotes the contaminant concentration in sediment, and *y* (μg/kg) denotes the contaminant concentration in fish tissues. *t*_1/2_ = 45, *t*_1/2_ = 90, and *t*_1/2_ = 180 represent LMG half-lives of 45, 90, and 180 d, respectively.
