# Supplementary material for: A Predictive Model for the Migration and Bioaccumulation of Residual Malachite Green in Aquaculture Sediments: Simulation and Validation
Source: Toxics. 2026 Sep 7;14(9):794. doi: 10.3390/toxics14090794 (PMC13611529; doi:10.3390/toxics14090794)
Supplement: Supplementary file 1 [file toxics-14-00794-s001.zip › toxics-4507977-supplementary.pdf]

Text S1. The methods of conventional culture simulation

The experiment was conducted as follows. A total of 10,000 healthy mandarin fish juveniles, each weighing approximately 10–20 g, were introduced into the MG-positive pond. The juveniles were supplied by a mandarin fish hatchery producer in Gaoming District, Foshan City, Guangdong Province, China, and were fed MG-free bait fish. Samples were collected at 0.1, 1.0, 2.0, 5.0, 10, 15, 20, 30, 40, 50, 70, 90, 120, 150, 180, and 210 d after the fish were introduced. The MG and LMG residues in the fish were determined at each sampling time, and sampling continued until neither MG nor LMG was detected in the fish.

Six replicate samples were collected at each sampling time, with each sample containing at least six fish. The heads, tails, viscera, and bones were removed from the fish, and the remaining tissues were thoroughly homogenized using a high speed homogenizer. The homogenized samples were stored at −20 °C until analysis.

Text S2. The half-life equation of LMG

When $t_{1/2}$ = 45 d,

$$C_t = 227.48 \times e^{(-0.0154 \times t)} \quad (20)$$

When $t_{1/2}$ = 45 d,

$$C_t = 227.48 \times e^{(-0.0063 \times t)} \quad (20)$$

When $t_{1/2}$ = 45 d,

$$C_t = 227.48 \times e^{(-0.0039 \times t)} \quad (20)$$

Table S1. Residual LMG concentrations in sediment and fish tissues under different half-life scenarios.C_S (μg/kg) denotes the contaminant concentration in sediment, and y (μg/kg) denotes the contaminant concentration in fish tissues. $t_{1/2}$ = 45, $t_{1/2}$ = 90, and $t_{1/2}$ = 180 represent LMG half-lives of 45, 90, and 180 d, respectively.

t(d)	Cs(μg/kg) ($t_{1/2}$ = 45)	y(μg/kg) ($t_{1/2}$ = 45)	Cs(μg/kg) ($t_{1/2}$ = 90)	y(μg/kg) ($t_{1/2}$ = 90)	Cs(μg/kg) ($t_{1/2}$ = 180)	y(μg/kg) ($t_{1/2}$ = 180)
1	224.00	0.1272	225.73	0.1282	226.59	0.1286
3	217.21	0.1814	222.28	0.1857	224.83	0.1878
5	210.62	0.1873	218.88	0.1947	223.08	0.1984
10	195.01	0.1878	210.62	0.2029	218.78	0.2107
15	180.56	0.1875	202.66	0.2105	214.55	0.2229
30	143.32	0.1867	180.55	0.2352	202.36	0.2636
45	113.76	0.1858	160.85	0.2627	190.86	0.3117
60	90.29	0.1848	143.30	0.2933	180.02	0.3685
90	56.89	0.1828	113.74	0.3654	160.14	0.5145
120	35.84	0.1805	90.27	0.4546	142.46	0.7173
150	22.58	0.1779	71.65	0.5645	126.73	0.9984
180	14.23	0.1194	56.87	0.4771	112.74	0.9459
210	8.96	0.0928	45.14	0.4671	100.29	1.0379
240	5.65	0.0721	35.83	0.4572	89.21	1.1385
270	3.56	0.0560	28.43	0.4473	79.36	1.2486
300	2.24	0.0435	22.57	0.4376	70.60	1.3688
330	1.41	0.0337	17.91	0.4278	62.81	1.5001
360	0.89	0.0262	14.22	0.4181	55.87	1.6432
390	0.56	0.0203	11.28	0.4085	49.70	1.7993
420	0.35	0.0157	8.96	0.3989	44.21	1.9692
450	0.22	0.0122	7.11	0.3893	39.33	2.1540
